# A Bayesian multivariate approach to estimating the prevalence of a superordinate category of disorders

**DOI:** 10.1002/mpr.1742

**Published:** 2018-09-14

**Authors:** Jonathan M. Fawcett, Nichole Fairbrother, Emily J. Fawcett, Ian R. White

**Affiliations:** ^1^ Department of Psychology Memorial University of Newfoundland St. John's NL Canada; ^2^ Department of Psychiatry and the Island Medical Program University of British Columbia Vancouver BC Canada; ^3^ Student Wellness and Counselling Centre Memorial University of Newfoundland St. John's NL Canada; ^4^ MRC Clinical Trials Unit University College London London UK

**Keywords:** Bayesian modelling, epidemiology, methods, multivariate model, meta‐analysis

## Abstract

**Objective:**

Epidemiological research plays an important role in public health, facilitated by the meta‐analytic aggregation of epidemiological trials into a single, more powerful estimate. This form of aggregation is complicated when estimating the prevalence of a superordinate category of disorders (e.g., “any anxiety disorder,” “any cardiac disorder”) because epidemiological studies rarely include all of the disorders selected to define the superordinate category. In this paper, we suggest that estimating the prevalence of a superordinate category based on studies with differing operationalization of that category (in the form of different disorders measured) is both common and ill‐advised. Our objective is to provide a better approach.

**Methods:**

We propose a multivariate method using individual disorder prevalences to produce a fully Bayesian estimate of the probability of having one or more of those disorders. We validate this approach using a recent case study and parameter recovery simulations.

**Results:**

Our approach produced less biased and more reliable estimates than other common approaches, which were at times highly biased.

**Conclusion:**

Although our approach entails additional effort (e.g., contacting authors for individual participant data), the improved accuracy of the prevalence estimates obtained is significant and therefore recommended.

## 1 INTRODUCTION

Epidemiology contributes to public health by characterizing the distribution of disorders as a means of informing public policy and optimally allocating resources (Oleckno, 2008). Meta‐analysis plays an important role (Fiest, Pringsheim, Patten, Svenson, & Jetté, 2014) by aggregating studies into more powerful estimates and modelling heterogeneity amongst samples (e.g., Chan et al., 2013; Fawcett, Fawcett, & Mazmanian, 2016; Russell, Fawcett, & Mazmanian, 2013; Simpson, Blizzard, Otahal, Van der Mei, & Taylor, 2011). Bayesian techniques are often used due to their flexibility and capacity to produce a credible estimate of beliefs given data from multiple sources (e.g., Ades & Sutton, 2006; Greenland, 2006).

Whereas aggregating individual disorder estimates is routine, it is unclear how to model the prevalence of superordinate categories—such as “any anxiety disorder” or “any cardiac disorder”—operationalized by combining multiple underlying conditions. We focus on an example from mental health, but our methods apply to any superordinate category. Within the health professions, superordinate categories play an important role by easing the interpretation and categorization of related symptoms and simplifying the identification of at‐risk populations without becoming lost in the minutiae of individual disorders. They also serve important social functions. For example, nonexperts better comprehend broad statements (one in five women suffer from anxiety disorders) than more specialized statements (one in 10 women suffer from social anxiety). Superordinate estimates therefore help generate the “big picture” within which public campaigns are most effective.

One challenge is that the category itself is often operationalized differently across the literature. For example, studies of anxiety disorders often include only a subsample of the eligible disorders—because they are the focal target or because a thorough examination of the individual disorders is too intensive. Prevalence might therefore be defined in one study as the probability of having panic disorder or agoraphobia but in another study as the probability of having social phobia or obsessive–compulsive disorder. This represents the “apples and oranges” problem described in most textbooks (e.g., Borenstein, Hedges, Higgins, & Rothstein, 2009, p. 379): If the underlying construct varies to such a degree between studies, their aggregation rests on questionable grounds.

The dominant solution has been to ignore variation in the disorders measured (e.g., Goodman, Watson, & Stubbs, 2016; Guo et al., 2016) or to include the number of disorders measured as a predictor (e.g., Baxter, Scott, Vos, & Whiteford, 2013; Steel et al., 2014). Although straightforward, either approach is likely to produce poor estimates. The exclusion of individual disorders in a category‐level estimate for a given study should negatively bias category prevalence. Including the number of disorders as a predictor and generating the prevalence of a study measuring all disorders may mitigate estimation bias, but this approach assumes that eligible disorders are equally prevalent—meaning that an increase of one disorder always influences the category‐level prevalence in the same manner. Although potentially true in some cases, it is often a questionable assumption.

An alternative approach is to perform a multivariate meta‐analysis of the individual disorders accounting for the fact that disorder prevalences are likely to be correlated both within and between studies (Jackson, Riley, & White, 2011). However, current multivariate approaches cannot estimate the prevalence of a superordinate category. This paper proposes a novel multivariate Bayesian approach that estimates the prevalence of a superordinate category while providing a more complete picture of its constituent disorders. We avoid aggregating prevalence estimates that vary in their operationalization, and instead model the prevalence estimates pertaining to the individual disorders and their interrelations. These parameters can be used to estimate the probability of having at least one of those disorders. In the following sections, we describe and then validate our model using a case study and parameter recovery simulations.

## 2 METHODS

### 2.1 Model

Put simply, our model uses aggregate and individual participant data (IPD) to estimate the prevalence of each individual disorder and the IPD to estimate the comorbidity between disorders. This portion of our model is implemented using Version 2.16 of the *Stan* modeling language (Stan Development Team, 2016) based on a binomial likelihood with a probit link function to estimate the mean prevalence and variability of each disorder across studies. Relevant code is available in Appendix A. In that appendix, Model 1 applies in the absence of IPD and assumes that the probit‐transformed latent correlations between the disorders are known. Model 2 uses IPD to estimate the probit‐transformed latent correlations between the disorders, incorporating uncertainty in these values into model estimates. In either case, we use those parameters to simulate a large sample of subjects from which to estimate the prevalence of having at least one disorder. This is a separate step conducted using the R programming language.

We define *μ*_*s,d*_ to be the probit‐transformed prevalence of disorder *d* in study *s*, and ***μ***_*s*, *_ = (*μ*_*s*, 1_, …, *μ*_*s*, *D*_) to be the vector of all probit‐transformed prevalences in study *s*. For the between‐study portion of our model, we assume that *μ*_*s,d*_ follows a normal distribution with mean *θ*_*d*_ and standard deviation *τ*_*d*_, and ***μ***_*s*, *_ follows a multivariate normal distribution with correlations given by matrix *ω*_*B*_. The calculations were implemented using a Cholesky factor decomposition of the correlation matrix to improve sampling efficiency, with a uniform LKJ prior (Lewandowski, Kurowicka, & Joe, 2009) for the Cholesky decomposition of *ω*_*B*_ (see the *Stan Reference Manual*). Thus, we have the following:

$$\mu_{s,*}∼\mathrm{MVN}\left(\theta_{*},\Sigma_{B}\right),\Sigma_{\mathrm{Bd}d^{\prime }}=\tau_{d}\times \omega_{\mathrm{Bd}d^{\prime }}\times \tau_{d^{\prime }},\text{Chol}\left(\omega_{B}\right)∼\mathrm{LKJ}\;\left(2\right).$$

Predictors could be incorporated by adding an additional term β_*_x_*s*_ to θ_*_ where *x*_*s*_ is the predictor value in study *s*.

For the probit‐transformed population prevalences (*θ*_*d*_), we used a mildly informative prior based on expert opinion. Because our case study models rare disorders, we used a normal distribution *θ*_*d*_ ∼ *N*(−1.88, 0.30^2^),implying that population prevalences Φ(*θ*_*d*_) ranging from 0.6% to 10.0% would be considered probable (within 2 *SD*) for any given disorder. For the between‐study estimates of standard deviation for each disorder (*τ*_*d*_), we employed a half‐normal distribution (truncated at 0) *τ*_*d*_ ∼ *N*(0,0.25^2^) with a location parameter equal to 0 and a standard deviation of 0.25, such that values larger than 0.5 would be unlikely. Presuming a mean prevalence of 3% for a given disorder, this would mean that 95% of “true” study‐specific prevalences lie between 0.2% and 18.9%; we felt this to be a reasonable range that balances the influence of the prior with convergence. Models using broader priors on *τ*_d_ produced similar results, albeit with less shrinkage, but did not converge as readily and were characterized by greater uncertainty in *τ*_d_ due to the relatively small amount of data available for any given disorder.

The within‐study model is broken into two parts—one dealing with the aggregate data and the other dealing with the IPD. For studies where the IPD were unavailable, the number of participants with a given disorder (*n*_*s,d*_) was modeled as arising independently from a binomial distribution with sample size *N*_*s*_ equal to the sample size of study *s* and probability defined earlier:

$$n_{s,d}∼\text{Binomial}\left(\Phi \left(\mu_{s,d}\;\right),N_{s}\right).$$

For studies where the IPD were available, prevalence was estimated using individual participant diagnoses. The binary (0 = *no diagnosis*, 1 = *diagnosis*) outcomes for each combination of participant and disorder were modelled as dichotomizations of an underlying multivariate normal distribution, with a threshold at 0 and a latent correlation matrix representing the relative comorbidity between disorders (***ω***_*C*_), to which we again applied an LKJ prior. Parameters for disorders not reported by that study were imputed within the model itself. Here, we define $\tilde{y}$_*s,d,i*_ as the underlying trait giving rise to participant *i* in study *s* suffering from disorder *d* whereby *y*_*s,d,i*_ as the observed outcome.

$$y_{s,d,i}=\left({\tilde{y}}_{s,d,i}>0\right),{\tilde{y}}_{s,*,i}∼\mathrm{MVN}\left(\mu_{s,*},\omega_{C}\right),\text{Chol}\left(\omega_{C}\right)∼\mathrm{LKJ}\left(2\right).$$

We fit the model using *Stan* but estimate superordinate category prevalence using a script built in *R 3.3.1* (R Core Team, 2016; sample code provided in Appendix B). Within this script, we first use the posterior distribution from our model to generate a large number of hypothetical participants, each taken from a different hypothetical study that is also generated from the posterior distribution; we do this by drawing 5,000 values from the posterior of ***μ***_*s*,*_ and then drawing a single participant ( ${\tilde{y}}_{s,*,i}$ and therefore *y*_*s*,*,*i*_) for each. This procedure makes use of the within‐study correlations between the disorders (***ω***_*C*_) estimated from the IPD (if present) or other sources (e.g., estimated elsewhere or arbitrarily assumed). The rows (*i*) of the resulting matrix represent individual hypothetical participants whereas the columns (*d*) represent individual disorders and each binary value indicates whether hypothetical participant *i* was diagnosed with disorder *d*. These binary values are summed for each row to simulate the number of diagnoses for a given hypothetical participant, with the overall prevalence representing the proportion of hypothetical participants with one or more disorder. This approach estimates the probability of a participant suffering from one or more AD while propagating our uncertainty across parameters, including between‐study heterogeneity (*τ*_*d*_). Prediction intervals for a new study are calculated in the same manner, with the exception that only a single “true” prevalence is estimated for each posterior sample (i.e., 5,000 hypothetical participants drawn from the same hypothetical study).

### 2.2 Implementation and sampling parameters

*Stan* is based on a variant of Markov Chain Monte Carlo sampling and is described in greater detail elsewhere (Hoffman & Gelman, 2014). Each model employed four independent chains. For the case study, chains included 5,000 iterations minus a warm‐up period of 2,500 resulting in 10,000 usable samples; for the simulations, chains included 1,000 iterations minus a warm‐up period of 500 resulting in 2,000 usable samples. We recommend the former but reduced this number to make the simulations tractable; fewer samples in our simulations should at worst handicap our estimates. Convergence was tested via visual inspection and using the *R‐hat* statistic (in all cases *R‐hat* ≈ 1 and *N*_*Effective*_ > 200, indicating convergence; Gelman & Hill, 2007). We report all parameters in terms of their median value as well as their highest density interval (HDI; Kruschke, 2014).

## 3 RESULTS

### 3.1 Case study of peripartum anxiety

#### 3.1.1 Case study description and parameters

**Figure 1:**
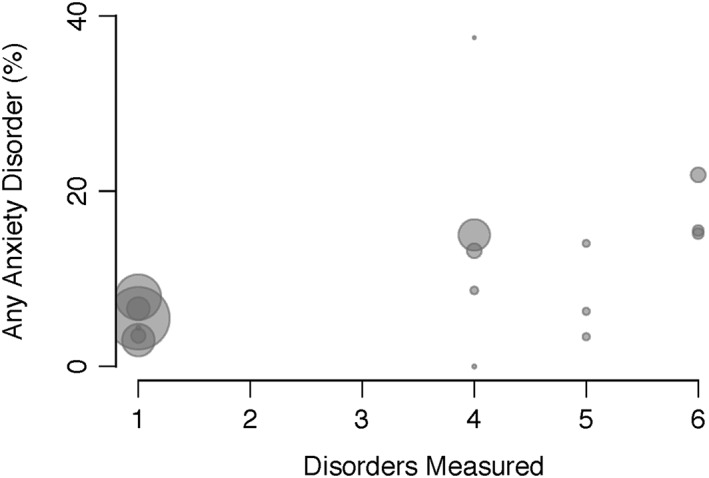
Prevalence (%) of “any” (i.e., having at least one) anxiety or related disorder (panic disorder, obsessive‐compulsive disorder, generalized anxiety disorder, social phobia, specific phobia, and posttraumatic stress disorder) plotted against the number of disorders that were measured in that sample. Marker size represents the relative sample size pertaining to each point, ranging from *N* = 24 to *N* = 2,202

We first illustrate our model using 10,033 participants from 18 articles reporting prevalence estimates for at least one of six selected anxiety and related disorders (AD; panic disorder, obsessive–compulsive disorder, generalized anxiety disorder, social phobia, specific phobia, and/or posttraumatic stress disorder; remaining AD are omitted for the sake of exposition). These data are summarized in Table 1. Of these data, we retrieved IPD for 1,506 participants (Chaudron & Nirodi, 2010; Fadzil et al., 2013; Fairbrother, Janssen, Antony, Tucker, & Young, 2016; Fisher, Wynter, & Rowe, 2010; Matthey & Ross‐Hamid, 2011; Usuda et al., 2016; Zar, Wijma, & Wijma, 2002). The average number of disorders measured by a given study was 3.4; Figure 1 depicts the percentage of participants diagnosed with at least one disorder as a function of the number of disorders measured. These studies represent a partial sample from a meta‐analysis of AD within peripartum populations (Fawcett, Fairbrother, Cox, White, & Fawcett, 2018). A partial sample was selected for illustration purposes as it allowed us to focus on the methodology itself rather than becoming lost in the details of the included articles; for this reason, it is important that the present analyses be used for demonstration purposes only. All but two studies (Fisher, Tran, Kriitmaa, Rosenthal, & Tran, 2010; Wenzel, Haugen, Jackson, & Brendle, 2005) permitted calculation of a superordinate (i.e., “any disorder”) prevalence estimate—representing the probability of having at least one of the disorders provided above; these studies were excluded from Section 3.1.2 but included in Section 3.1.3. They were selected despite the missing “any disorder” prevalence estimates to highlight the fact that our model makes better use of the available data.

**Table 1:** Descriptive statistics and prevalence estimates (%) reported by each study included in the case study

First author	Year	*N*	Prevalence any	Panic disorder	OCD	GAD	Social phobia	Specific phobia	PTSD
Zar*	2002	453	21.9	1.3	0.2	0.9	2.7	18.3	1.3
Wenzel	2003	68	4.4	–	–	4.4	–	–	–
Wenzel	2005	147	–	1.4	2.7	8.2	4.1	–	–
Uguz	2007	434	3.5	–	3.5	–	–	–	–
Rogal	2007	1100	3.0	–	–	–	–	–	3.0
Mota (Preg.)	2008	451	13.2	2.1	–	1.9	3.3	9.3	–
Mota (Post.)	2008	1061	15.0	4.0	–	2.3	2.5	10.2	–
Seng	2009	1581	7.9	–	–	–	–	–	7.9
Kersting	2009	65	0.0	0.0	0.0	–	–	0.0	0.0
Fisher (Preg.)	2010a	199	–	1.5	–	10.6	–	–	–
Fisher (Post.)	2010a	165	–	4.2	–	11.5	–	–	–
Chaudron*	2010	24	37.5	4.2	29.2	–	–	8.3	8.3
Fisher*	2010b	196	8.7	0.0	–	2.6	3.6	3.6	–
Uguz	2010	309	15.5	1.9	5.2	3.6	3.2	3.2	0.0
Matthey*	2011	171	14.0	2.9	2.9	11.1	4.1	–	0.6
Prenoveau	2013	2202	5.5	–	–	5.5	–	–	–
Fadzil*	2013	175	6.3	5.7	0.0	0.0	0.6	–	0.0
Kim	2014	745	6.6	–	–	–	–	–	6.6
Usuda*	2016	177	3.4	1.1	1.7	0.0	1.1	–	0.6
Fairbrother*	2016	310	15.2	0.7	3.6	3.2	5.2	7.4	0.7

*Note*. Prevalence estimates from Matthey and Ross‐Hamid (2011) were calculated from raw data inclusive of additional subjects beyond those reported in their article. Studies contributing IPD are marked with an asterisk (*) and “–” represent values that were not reported. IPD: individual patient data; Preg.: pregnant group; Post.: postpartum group; OCD: obsessive–compulsive disorder; GAD: generalized anxiety disorder; PTSD: posttraumatic stress disorder.

#### 3.1.2 Univariate random‐effects models using “any disorder” estimates

We first analyzed the “any disorder” prevalences, using a series of univariate random‐effects models meant to emulate current practice. In keeping with how these analyses have been conducted in the past, we logit‐transformed the estimates for each study and aggregated them using the *rma* function from the *metafor* package (Viechtbauer, 2010). We fit this model twice—once to the full data set, including studies measuring any number of disorders, and again including only studies measuring three or more disorders. All analyses are summarized in Table 2. Clearly, requiring the inclusion of at least half of the measured disorders increased the estimated prevalence. This might suggest that researchers should require a minimum number of disorders for inclusion; however, the only logical criterion would require inclusion of all disorders. Unfortunately, such a criterion would exclude most of the extant data; only three studies (Fairbrother et al., 2016; Uguz, Gezginc, Kayhan, Sarı, & Büyüköz, 2010; Zar et al., 2002) in the current sample provided “any disorder” prevalence estimates inclusive of all six disorders.

**Table 2:** Prevalence estimates from the case study for each estimation procedure

Method	No. of samples	Disorder	Prev.	τ_Probit_	τ_Logit_
BMV‐IPD	20 (7 IPD)	Any	19 [15, 23]	–	–
		Panic	2 [1, 3]	.26 [.11, .45]	–
		OCD	3 [1, 5]	.47 [.26, .71]	–
		GAD	4 [2, 6]	.41 [.26, .60]	–
		Social Phobia	3 [2, 4]	.11 [.00, .24]	–
		Specific Phobia	6 [3, 9]	.36 [.19, .58]	–
		PTSD	2 [1, 3]	.47 [29, .70]	–
					
FRE	17	Any	9 [6, 13]	.48	.77
FRE‐H	11	Any	13 [9, 18]	.40	.64
FRE‐A	3	Any	18 [14, 23]	.14	.22
FRE‐M	17	Any	16 [10, 23]	.33	.53
FRE‐MH	11	Any	14 [7, 25]	.44	.70

*Note*. Individual disorder estimates are also provided for our preferred multivariate approach. Estimates of *τ* were calculated in probit‐space for the multivariate Bayesian approach and in logit‐space for the univariate Frequentist approaches; for comparison, the logit values were converted into approximate probit space via division by 1.6. BMV‐IPD: Bayesian multivariate w/individual patient data; FRE: Frequentist univariate; FRE‐H: as FRE but including studies reporting at least three disorders; FRE‐A: as FRE but including studies reporting six disorders; FRE‐M: Frequentist univariate w/predictor; FRE‐MH: as FRE‐M but including studies reporting at least three disorders.

Another approach is to use the number of measured disorders to predict the prevalence of a hypothetical study measuring all disorders (e.g., Baxter et al., 2013). We again fit this model twice, once including all studies and again including only studies measuring three or more disorders. This approach predicts a higher prevalence estimate but is unstable (see Sections 3.1.2 and 3.1.3). Importantly, whereas the effect of number of disorders was significant in the former model (*P* < 0.001), it was not significant in the latter model (*P* = 0.693)—likely owing to reduced statistical power combined with range restriction. This would be worsened should the number of disorders be treated categorically (e.g., 1–2 disorders and 3–4 disorders;e.g., Baxter et al., 2013) because converting continuous predictors into categorical ones reduces statistical power (Gelman & Park, 2009).

#### 3.1.3 Bayesian multivariate models

We next applied our model to the same data using the approach described in Sections 2.1 and 2.2. From the posterior of this model, we calculated the probability of having one or more, two or more, and so forth disorders. As depicted in Table 3, this estimate is close to those produced by either the univariate analysis of studies measuring all disorders or the initial regression model. However, the HDI is equivalent to or narrower than the confidence intervals from any of the previous models—even though Bayesian confidence intervals for aggregate effects are generally broader than their Frequentist counterparts because the former take uncertainty in *τ* into account (in this case for each disorder) whereas the latter assume that *τ* is known. This reflects—in part—the greater ability of our Bayesian model to make use of all data.

**Table 3:** Probability of having at least 1, 2, 3, or 4 anxiety or related disorders (AD) within our case study as fit using a multivariate Bayesian model with IPD (Model 2)

	Number of disorders			
	1+	2+	3+	4+
Prevalence	19% (15%, 23%)	5% (3%, 7%)	1% (1%, 2%)	0% (0%, 1%)
Prediction Interval	(7%, 34%)	(1%, 10%)	(0%, 3%)	(0%, 1%)

*Note*. The estimated probability of having 5+ disorders was negligible and therefore excluded. Prevalence estimates refer to the prevalence within a “typical” study whereas the prediction intervals indicate instead the range of credible values estimated from a new study similar to those included in the model.

**Figure 2:**
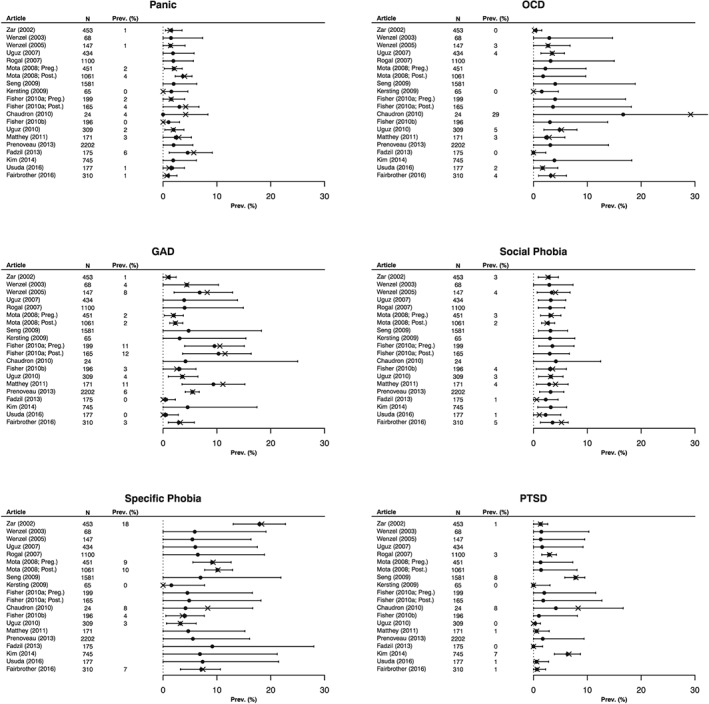
Prevalence (%) for each anxiety or related disorder (panic disorder, obsessive‐compulsive disorder, generalized anxiety disorder, social phobia, specific phobia, and posttraumatic stress disorder) and sample within our case study, simulated based on sample size and parameter estimates derived from our model. Circles represent median prevalence estimates, and error bars represent HDI_95%_; the reported prevalence for each study is denoted by an “X.” Predictions are provided for studies not measuring a given disorder, representing the probable prevalence of that disorder within that sample. These studies can be identified by the absence of an “X” in the plot and the absence of a numerical prevalence value in the third column

**Figure 3:**
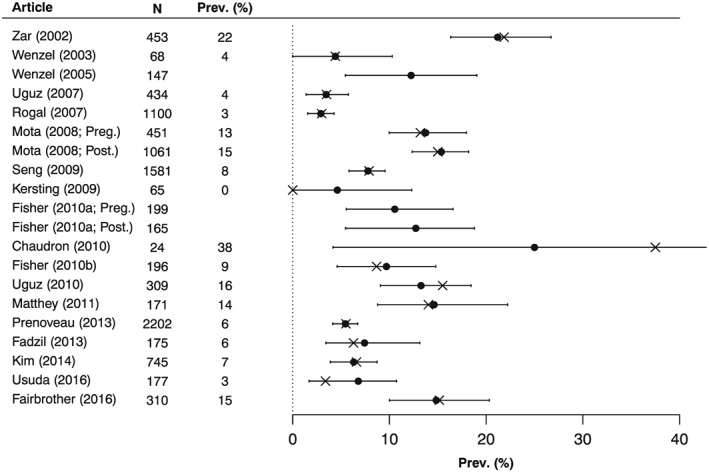
Prevalence (%) of having at least one anxiety or related disorder (panic, obsessive‐compulsive disorder, generalized anxiety disorder, social phobia, specific phobia, and posttraumatic stress disorder) for each study within our case study simulated based on sample size and parameter estimates derived from our model. Circles represent median prevalence estimates and error bars represent HDI_95%_; the reported prevalence for each study is denoted by an “X.” Predictions are provided for studies for which the “any disorder” prevalence was unavailable, representing the probable prevalence of having at least one disorder within that sample. These studies can be identified by the absence of an “X” in the plot and the absence of a numerical prevalence value in the third column.

We next examined how well our model captured the data. Figure 2 depicts observed and predicted disorder prevalence for each study. All estimates fall well within the boundaries of the model predictions. We also generated “any disorder” predictions for each study in the same manner, with the “any disorder” prevalence defined as the probability of having at least one of the disorders *measured* in that particular study. These predictions are provided in Figure 3 alongside the reported “any disorder” prevalence values, where available. Despite these data not entering our model, they are nonetheless well represented by the model's predictions.

The above analyses made use of mildly informative priors based on expert knowledge. We repeated the analysis using less informative (hence less realistic) priors. The uninformative prior placed on mean prevalence estimates was based on a normal distribution with a mean of −1.88 and a standard deviation of 1, calibrated such that mean prevalences ranging from <0.1% to 54.8% would be considered probable for any given disorder. For the between‐study estimates of standard deviation for each disorder (*τ*_*d*_), the *SD* of the half‐normal was increased to 0.35, meaning that values higher than 0.70 would be uncommon. Presuming a mean prevalence of 3% for a given disorder, a probit‐transformed standard deviation of 0.70 means that the “true” prevalence within any given study might vary credibly from <0.1% to 31.6%. Despite these unrealistic prior expectations, the model output remained largely unchanged—producing an overall estimate of 20%, HDI_95%_ (15%, 26%). Although the HDI has increased in size, this is because the priors allocated credibility to the possibility that as many as 80% of the participants in a typical study suffered from a given disorder, increasing uncertainty in *τ*_*d*_.

### 3.2 Parameter recovery simulations

#### 3.2.1 General method

We next conducted three parameter recovery simulations comparing our model against the approaches described in Section 3.1.2. The first is based on the case study with respect to the number of samples, disorders measured, participants per sample, and so forth. The second and third explore how different levels of between‐study variability influence performance across estimation approaches. Each simulated dataset contained 20 studies with sample sizes randomly drawn from an exponential distribution with a rate of .005 to which 100 was added. A measurement process simulated the tendency for studies not to measure all disorders by assigning each disorder a probability for inclusion in that study. To ensure all disorders were represented, the first two studies in each simulation were exempt from this measurement process. The probability of having one or more of the measured disorders was then calculated for each study given the disorders measured; these values were used to test alternate estimation approaches. The first seven studies in any given simulation provided IPD.

For our first simulation, we generated data like the case study provided in Section 3.1.1. We used the median ***ω***_*C*_ and ***ω***_*B*_ matrices from that model to emulate a realistic distribution of interdependencies. Similarly, the “true” prevalences for each disorder were set at 1.5%, 2.0%, 2.5%, 3.5%, 4.0%, and 6.0%. Probit‐transformed heterogeneity (*τ*) was derived from the values estimated in Section 3.2 (rounded to 0.50, 0.25, 0.45, 0.10, 0.40, and 0.35) and the measurement probabilities were set to 50%, 50%, 70%, 50%, 70%, and 40%.

Our second and third simulations explored the effect of heterogeneity by changing *τ* to 0.1 (low heterogeneity) or 0.4 (high heterogeneity) for all disorders. In both cases, we also made “true” prevalences more diverse than in our case study (1.0%, 2.0%, 3.0%, 5.0%, 7.0%, and 9.0%). Our goal in doing so was to evaluate how the predictor approach would perform when the assumption of equal prevalence across disorders was violated.

For each simulated data set, the prevalence of having one or more of the individual disorders was estimated using the univariate approaches presented in Section 3.1.1 (e.g., Baxter et al., 2013; Goodman et al., 2016). We also used our Bayesian approach, first assuming that the between‐disorder (***ω***_*C*_) correlation matrix was known (Model 1), then estimating this matrix from independent participant data (Model 2). We fit the following to each simulated sample:

- **FRE:** Frequentist random effects model fit to the “any disorder” prevalences ignoring the number of disorders measured;
- **FRE‐H:** as FRE but only including studies that reported at least half of the disorders;
- **FRE‐M:** Frequentist random effects model fit to the “any disorder” prevalences including the number of disorders measured as a predictor and estimating the prevalence of a study measuring all disorders;
- **FRE‐MH:** as FRE‐M but only including studies which reported at least half of the disorders;
- **BMV‐K:** Bayesian multivariate approach fit to the individual disorder estimates, using only aggregate data and assuming ***ω***_*C*_ is known (Model 1);
- **BMV‐IPD:** Bayesian multivariate approach fit to the individual disorder estimates, using both aggregate and IPD and estimating ***ω***_*C*_ from the model (Model 2);

For each model including a predictor, we also recorded the associated *P* value. We ran each simulation 100 times—with every iteration representing a simulated meta‐analysis. Iterations required ~6 hr each. For each simulation study, the “true” prevalence of having at least one disorder was estimated by simulating IPD for 10,000 participants—each from a separate study—10,000 times, calculating the overall prevalence for each and taking the median.

#### 3.2.2 Simulation results

**Figure 4:**
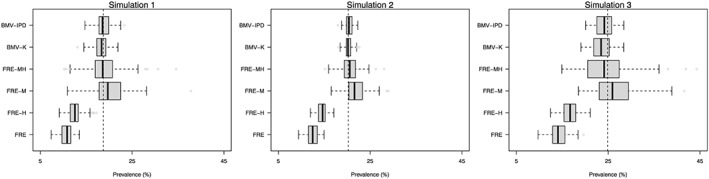
Prevalence (%) of having one or more disorder within each simulated meta‐analysis using each of the estimation approaches described in text for Simulation 1 (τ and prevalence based on case study), Simulation 2 (τ = 0.1),s and Simulation 3 (τ = 0.4); the “true” prevalence is represented by a dotted line within each plot

Each panel of Figure 4 depicts the prevalence of having at least one disorder as estimated by each procedure within each simulation. The “true” prevalence for each is depicted by a dotted line; these “true” prevalences depend on individual disorder heterogeneity and hence differ across simulations.

Neither ignoring the number of disorders in each study nor including them as a predictor is adequate. Ignoring variation in the number of measured disorders tended to catastrophically underestimate the “true” prevalence. This underestimation was mildly improved by requiring that a nominal number of disorders be measured for inclusion; we included as our cutoff the midpoint of the number of disorders measured, but this procedure should approach an unbiased estimate as the required number of disorders approaches the total number of possible disorders. Imposing this limitation necessarily decreases the number of studies available for analysis, making it inefficient. Including the number of disorders as a predictor resulted in an unbiased but variable estimate. A slight positive bias emerged in Simulations 2 and 3, where the prevalences differ from one another to a greater degree, and the variability of the estimate scaled with increasing heterogeneity.

In contrast, the Bayesian models produced efficient estimates with no discernable bias when heterogeneity was moderate or low (Simulations 1 and 2), with a slight negative bias for BMV‐K (median bias of approximately −1%) when heterogeneity was high (Simulation 3). This bias is slight, so we do not wish to over‐interpret; nonetheless, we speculate that it arises because all values of *τ* within this simulation are near the upper range of our prior. Therefore, a small amount of shrinkage is expected. Estimation of *τ* has consequences for the overall prevalence in part because of the use of a normal distribution on the probit scale: for a fixed probit‐transformed prevalence of less than 0 (corresponding to prevalences below 50%), increasing *τ* increases both left and right tails on the probit scale, but this has a greater impact for the right tail on the probability scale. As a result, larger *τ* means larger prevalence (assuming *μ* is unchanged). Inspection of the posterior estimates across our simulations supports this conviction—with the *τ* for each disorder being underestimated on average by 0.05 (results not shown). Such underestimation is negligible for individual disorders but could aggregate to produce a slight bias overall. BMV‐IPD was unaffected.

Our model is further supported by coverage estimates and confidence interval widths provided in Table 4. These estimates represent the percentage of samples wherein the confidence or highest density intervals included the “true” prevalence value. The predictor model and the Bayesian model maintained nominal coverage across all simulations (with the exception of BMV‐K in Simulation 3) whereas the simple univariate models performed comparatively poorly. Confidence interval widths from the predictor model were two to three times wider than from the other methods.

**Table 4:** Coverage statistics and mean confidence interval width (defined as the difference between upper bound and lower bound) within each simulated sample for each estimation approach and presented separately for Simulation 1 (*τ* and prevalence based on case study), Simulation 2 (*τ* = 0.1), and Simulation 3 (*τ* = 0.4)

	Coverage (%)	Mean width (%)
Simulation 1		
FRE	0 (0)	6
FRE‐H	16 (4)	7
FRE‐M	95 (2)	17
FRE‐MH	94 (2)	17
BMV‐AG	97 (2)	7
BMV‐IPD	98 (1)	7
Simulation 2		
FRE	0 (0)	5
FRE‐H	2 (1)	5
FRE‐M	95 (2)	11
FRE‐MH	99 (1)	10
BMV‐AG	99 (1)	4
BMV‐IPD	100 (0)	4
Simulation 3		
FRE	0 (0)	8
FRE‐H	13 (3)	9
FRE‐M	99 (1)	22
FRE‐MH	96 (2)	22
BMV‐AG	89 (3)	8
BMV‐IPD	94 (2)	8

*Note*. Value in brackets represents the Monte Carlo error for that estimate; all Monte Carlo errors for mean width are less than 0.5% (maximum of 0.49% and minimum of 0.05%) and therefore would have rounded to 0.

Finally, for Simulation 1, the statistical power for testing the slope for the number of measured disorders was relatively high (86%) in the model inclusive of all studies but only moderate (52%) when studies were required to measure half of the disorders for inclusion. The same pattern was observed in Simulations 2 and 3, with the exception that power was universally high when heterogeneity was low (99% and 90% for FRE‐M and FRE‐MH, respectively) and more variable when heterogeneity was high (83% and 33% for FRE‐M and FRE‐MH, respectively).

Differences in the precision of estimates from FRE‐M and BMV‐IPD are larger in the simulations than in the case study. Because the BMV‐IPD approach uses individual disorder estimates, the sample on which our case study is based includes a preponderance of single disorder papers that are otherwise rare in this type of analysis (e.g., Goodman et al., 2016) where studies measuring multiple disorders are preferred. Our simulations match the overall mean number of disorders measured from the case study but do not specifically reflect this idiosyncratic quality. Single disorder estimates will be less variable (they are affected by heterogeneity from only a single disorder) and would also have a high degree of influence in the predictor analysis, stabilizing the slope and therefore increasing precision of the estimate. Additional simulations support this interpretation, showing that meta‐analyses forced to emulate the distribution of disorders measured from the case study produce more precise estimates for the predictor approaches due to reduced uncertainty in the slope (results not shown). Importantly, inclusion of so many single disorder estimates is uncommon and still rests on the assumption that disorders are equally prevalent and comorbid. Our model outperforms a purely predictor based model—even under these conditions—just not as drastically.

## 4 DISCUSSION

### 4.1 Advantages

#### 4.1.1 More efficient use of data

Our model permits the combination of more information than is possible with a univariate approach. For example, the inclusion of single disorder studies in a univariate analysis offers no specific advantage and is liable to bias aggregate estimates. In our model, single disorder studies inform the prevalence of the disorder in question—which in turn improves the precision of our category‐level estimate. Further, whereas most studies include individual disorder prevalence estimates—not all studies provide workable category‐level estimates, either due to inclusion of ineligible disorders (e.g., depression) or omission of the “any disorder” prevalence. Therefore, the number of studies eligible for inclusion is higher for our model, and the data obtained are put to better use.

#### 4.1.2 Access to an informative posterior distribution

Our model produces a posterior distribution representing the combination of all pertinent knowledge. In addition to prevalence estimates for the individual disorders, this posterior provides the probability of any arbitrary event or confluence of events. For example, Table 3 provides the probability of having any number of disorders. It is possible to use the same approach to explore comorbidity. If a clinician knew that a given participant had been diagnosed with panic disorder, they might like to know the odds of that participant suffering from generalized anxiety disorder. This can be calculated from the posterior using the conditional probability of having generalized anxiety disorder given a diagnosis of panic disorder. Based on the case study, this probability is 20%, HDI_95%_ (6%, 39%), and meaning that peripartum participants with panic disorder have a one in five chance of having generalized anxiety disorder. Prediction intervals representing the range of possible “true” values across the distribution of samples similar to those included in the present analysis can also be calculated (in the current case: HDI_PI95%_ [0%, 58%]). One could likewise choose to calculate the probability of having any number of specific disorders (e.g., What is the probability of having a diagnosis of generalized anxiety disorder, panic disorder, and social phobia?). The possible questions addressable using the output of this model are limited only by imagination and need.

#### 4.1.3 Easy handling of dependencies between samples

One common challenge that faces meta‐analysts is dealing with multiple samples from the same study or population; such estimates are dependent on one another in a manner that will artificially deflate uncertainty in the aggregate prevalence estimate if not addressed. For the Frequentist approaches described in Section 3, this dependency is often ignored; however, they are readily handled in our Bayesian approach—and in fact, complex random structure could be implemented. Our case study included two studies with multiple samples (Fisher, Tran, et al., 2010; Mota, Cox, Enns, Calhoun, & Sareen, 2008), which were handled by estimating a single “true” prevalence used by all samples from the study in question.

#### 4.1.4 Use of prior knowledge

Our approach incorporates expert knowledge into the model to improve estimation. As demonstrated earlier, the improvement in the precision of an estimate due to a reasonable (but skeptical) prior can be substantial. Given the availability of expert knowledge, this would seem to be an easy way to improve model efficiency.

### 4.2 Potential limitations

#### 4.2.1 Assumes access to IPD

The first limitation is that we assume access to IPD from which to estimate the within‐study correlations amongst the individual disorders (***ω***_*C*_). Without those data, our model must assume that ***ω***_*C*_ is known (Model 1), which is uncommon. Although gathering individual data can be difficult, the improved accuracy is worth the effort. This effort could be lessened if those reporting epidemiological studies shared their data or provided a table summarizing each participant suffering from at least one disorder, with a list of each diagnosis they received (e.g., 5 × panic disorder, 2 × panic disorder + posttraumatic stress disorder; e.g., Zar et al., 2002).

#### 4.2.2 Assumes homogeneity of within‐study correlations between disorders (**ω**_C_)

This assumption was made partially to simplify our model but also because we do not believe that IPD from a broad enough sample of studies is generally available to estimate heterogeneity amongst the correlations. We highlight this as a potential area for future development.

#### 4.2.3 Assumes studies are sampled from a larger population

Interpretation of any meta‐analysis—including those based on the current approach—is complicated by the presence of heterogeneity. This is because samples included in the model are assumed to be drawn randomly from a broader population of potential samples. Therefore, consideration must be given to the populations being studied, and the results must be interpreted in light of the *distribution* of prevalence estimates (e.g., using prediction intervals).

#### 4.2.4 Assumes generality based on a single application

Current findings suggest our model to be an improvement over existing approaches; however, these findings (including the parameter recovery simulations) are based on a particular application in the field of Psychology. Although we expect fully that our model will generalize to other topics, as with all new techniques, it is possible that the observed benefits may be linked to the circumstances of the selected case in unexpected ways. Future applications to other fields will determine whether this is true.

### 4.3 Alternative approaches and extensions

Although we demonstrate our multivariate Bayesian model to be in many ways superior to the univariate Frequentist models previously used to estimate the prevalence of a superordinate category (e.g., Goodman et al., 2016), there are certainly alternative approaches to address this problem. For example, one could use the expectation maximization algorithm to estimate the prevalence of each of the individual disorders, treating unmeasured disorders within a given sample as missing data. One could also implement a multilevel, multivariate Frequentist model (e.g., using *metafor*; Viechtbauer, 2010) to estimate the prevalence of the individual disorders. However, in either case, it would be necessary to combine the individual disorder estimates into an estimate of the superordinate category prevalence. This would most likely involve a simulation procedure similar to that described in Appendix B—and would necessitate inclusion of IPD to estimate the comorbidity across disorders. For that reason, we do not expect either solution to be simpler than the current approach and neither would not benefit from most strengths detailed earlier. In short, whereas other approaches may be preferable under certain circumstances, we believe that complex evidence synthesis often benefits from a Bayesian approach such as ours (e.g. Ades & Sutton, 2006).

Even so, the current model represents only an initial step towards developing a general method for estimating the prevalence of a superordinate category. For that reason, there are many possible extensions. As one representative example, our model could be modified to address bias caused by variation in measurement or selection across studies, perhaps using bias modelling methods such as those of Turner, Spiegelhalter, Smith, and Thompson (2009). This would permit quantification of—and adjustment for—biases due to variation in the quality of the included studies. This would reflect a clear improvement and is a potential target for future development.

## 5 CONCLUSION

Estimating the prevalence of a superordinate category of disorders based on studies with differing operationalization of that category is both common and ill‐advised (e.g., Baxter et al., 2013; Goodman et al., 2016; Guo et al., 2016; Steel et al., 2014). We propose instead a Bayesian model using IPD that we have shown to produce unbiased, efficient estimates where other approaches are biased and/or inefficient. The accurate estimation of disease prevalence is of profound clinical importance, because it serves to guide public policy. To use our case study as an example, a shift from 9% to 19% in the estimated prevalence of peripartum AD means a change from one in 10 to one in five peripartum women suffering from one or more AD. Such a shift has implications for the allocation of public funds and even screening procedures. For this reason, we believe that the current Bayesian approach will have real clinical importance and hope that the present article will encourage future meta‐analysts to adopt a similar approach when estimating superordinate category prevalence in the future.

## CONFLICTS OF INTEREST

The authors report no potential conflicts of interest.

## FUNDING/SUPPORT

This research received no direct financial support from any granting agency. J. M. F. was supported by an NSERC Discovery Grant and IRW was supported by the Medical Research Council (Unit Programme MC_UU_12023/21).

## Supporting information

Supporting info item

## Appendix 1 Appendix A: Model Code

Below is the *Stan* code necessary to fit Model 2 reported in text; this code can be extended to incorporate predictors by adding a vector of slopes to the parameter section that is then multiplied by the predictor and added to each of the disorders within the transformed parameters section (see commented lines). To fit Model 1 reported in text, simply delete all lines corresponding to the individual participant data model. If you have any comments or questions pertaining to this code, you should contact the first author (jmfawcet@gmail.com).

## Appendix 2 Appendix B: *R* code necessary to generate overall prevalence estimates

The *R* code necessary to generate the overall prevalence estimates of the models reported in Appendix B is included as a separate file.
